# High Resolution X Chromosome-Specific Array-CGH Detects New CNVs in Infertile Males

**DOI:** 10.1371/journal.pone.0044887

**Published:** 2012-10-09

**Authors:** Csilla Krausz, Claudia Giachini, Deborah Lo Giacco, Fabrice Daguin, Chiara Chianese, Elisabet Ars, Eduard Ruiz-Castane, Gianni Forti, Elena Rossi

**Affiliations:** 1 Unit of Sexual Medicine and Andrology, Molecular Genetic Laboratory, Department of Clinical Physiopathology, University of Florence, Florence, Italy; 2 Andrology Service, Fundació Puigvert, Barcelona, Spain; 3 Molecular Biology Laboratory, Fundació Puigvert, Universitat Autònoma de Barcelona, Barcelona, Spain; 4 Endocrinology Unit, Department of Clinical Physiopathology, University of Florence, Florence, Italy; 5 Biology and Medical Genetics, University of Pavia, Pavia, Italy; Temasek Life Sciences Laboratory, Singapore

## Abstract

**Context:**

The role of CNVs in male infertility is poorly defined, and only those linked to the Y chromosome have been the object of extensive research. Although it has been predicted that the X chromosome is also enriched in spermatogenesis genes, no clinically relevant gene mutations have been identified so far.

**Objectives:**

In order to advance our understanding of the role of X-linked genetic factors in male infertility, we applied high resolution X chromosome specific array-CGH in 199 men with different sperm count followed by the analysis of selected, patient-specific deletions in large groups of cases and normozoospermic controls.

**Results:**

We identified 73 CNVs, among which 55 are novel, providing the largest collection of X-linked CNVs in relation to spermatogenesis. We found 12 patient-specific deletions with potential clinical implication. Cancer Testis Antigen gene family members were the most frequently affected genes, and represent new genetic targets in relationship with altered spermatogenesis. One of the most relevant findings of our study is the significantly higher global burden of deletions in patients compared to controls due to an excessive rate of deletions/person (0.57 versus 0.21, respectively; p = 8.785×10^−6^) and to a higher mean sequence loss/person (11.79 Kb and 8.13 Kb, respectively; p = 3.435×10^−4^).

**Conclusions:**

By the analysis of the X chromosome at the highest resolution available to date, in a large group of subjects with known sperm count we observed a deletion burden in relation to spermatogenic impairment and the lack of highly recurrent deletions on the X chromosome. We identified a number of potentially important patient-specific CNVs and candidate spermatogenesis genes, which represent novel targets for future investigations.

## Introduction

Male factor infertility affects about 7% of men in the general population and the etiology of altered spermatogenesis remains unknown in about 40% of cases (“idiopathic infertility”) and it is likely that a large proportion of them are caused by still unknown genetic factors [1]. Nevertheless, besides abnormal karyotype and Y chromosome microdeletions no other recurrent genetic anomalies have been identified in men with primary testicular failure, raising questions about the appropriateness of the investigative approaches used so far [2]–[4]. The first innovative study applying whole-genome analysis of SNPs and the successive follow-up study failed in leading to the identification of recurrent genetic factors with large effect size [5], [6]. Recently, high resolution array Comparative Genomic Hybridisation (array-CGH) studies identified new spermatogenesis candidate genes on autosomes and on the X chromosome and some recurring and private patient-specific CNVs with potential clinical interest [7], [8].

Both sex chromosomes are enriched with genes prevalently or exclusively expressed in the testis [9], [10]. Nevertheless, only Y chromosome-linked Copy Number Variants (CNVs) and Y-linked genes have been demonstrated as important contributors to impaired sperm production in humans [for review see [11], [12]). In particular, the so called AZoospermia Factor (AZF) regions on the Yq have been found deleted in about 5–10% of azoospermic men (absence of spermatozoa in the ejaculate) and 2–5% of severe oligozoospermic men (<5 millions spermatozoa in the ejaculate). Data on the potential role of X-linked gene products in spermatogenesis derive mainly from model organisms and a higher than expected number of X-linked spermatogenesis genes have been identified [10],[13]. The apparent paucity of information in humans is probably related to the scarcity of X-linked genes studied (only eight), none of which yet described as causative, except for the *AR* gene [14]. Similarly, the question whether the X chromosome contains AZF-like regions has not been sufficiently explored so far.

In order to advance the understanding of the role of X-linked CNVs and genes in male infertility, we applied an innovative approach based on high resolution X chromosome specific array-CGH. Given that such a detailed analysis of the X chromosome has not been published until now and the testicular function of subjects included in the Genomic Variant Database is unknown (except for 30 X-linked CNVs (23 duplications and 7 deletions) reported in the recent paper by Tuttelmann et al. [7]), ours is the first study providing a detailed analysis of X-linked losses and gains in several hundred subjects with known sperm parameters.

## Materials and Methods

### Subjects

The local Ethical Committees of the University Hospital Careggi and the Fundació Puigvert approved the study. All participants signed an informed consent. We analyzed with array-CGH 96 idiopathic infertile subjects with different grade of spermatogenic impairment (49 azoospermic, 25 cryptozoospermic and 22 oligozoospermic men) and 103 normozoospermic men. Infertile patients were selected on the basis of a comprehensive andrological examination including medical history, semen analysis, scrotal ultrasound, hormone analysis, karyotype and Y chromosome microdeletion screening. Patients with mono- or bilateral cryptorchidism, varicocele grades 2 and 3, obstructive azoospermia, recurrent infections, iatrogenic infertility, hypogonadotrophic hypogonadism, karyotype anomalies, Y chromosome microdeletions including partial deletions of the *AZFc* region, and partial *AZFc* duplications and patients with non-Italian or non-Spanish origin were excluded. Testis histology was available for 47 men. Controls in the Spanish cohort were fertile normozoospermic men undergoing pre-vasectomy, whereas the Italian control cohort included normozoospermic volunteers not belonging to infertile couples (60% with proven fertility). The ethnic/geographic composition was similar in the control and patient groups (40% Spanish and 60% Italians).In the second part of the study, we performed a case-control association study reaching a total of 359 patients and 370 normozoospermic controls on 13 selected CNVs which appeared to be specific to infertile men based on the array-CGH analysis. Detailed phenotypic data relative to the study populations are provided in Table 1.

**Table 1:** Clinical description of the study population.

A			
SPERM COUNT		PATIENTS (n = 359)	CONTROLS (n = 370)
**Total sperm count (10^6^)**	median (25^th^–75^th^ percentile)	2.6 (0.00–13.62)	263.20 (159.00–405.50)
	mean ± SD	8.77±12.72	311.79±199.99
**Sperm concentration (10^6^/ml)**	median (25^th^–75^th^ percentile)	0.90 (0.00–4.40)	76 (50.00–117.50)
	mean ± SD	2.56±3.27	91.32±59.64

B				
GEOGRAPHIC ORIGIN	SEMEN PHENOTYPE	ARRAY-CGH STUDY (n = 96)	CASE-CONTROL STUDY (n = 263)	TOTAL (n = 359)
**ITALIAN (n = 233)**	azoospermic	15	42	57
	cryptozoospermic	20	17	37
	oligozoospermic	22	117	139
	*severe oligozoospermic (<5×10^6^/ml)*	*17*	*47*	*-*
	*moderate oligozoospermic(<20×10^6^/ml)*	*0*	*12*	*-*
**SPANISH (n = 126)**	azoospermic	34	15	49
	cryptozoospermic	5	36	41
	oligozoospermic	0	36	36
	*severe oligozoospermic (<5×10^6^/ml)*	*0*	*24*	*-*
	*moderate oligozoospermic(<20×10^6^/ml)*	*0*	*12*	*-*

C			
HORMONAL PARAMETERS	ARRAY-CGH STUDY (n = 96)	CASE-CONTROL STUDY (n = 263)	TOTAL (n = 359)
**FSH (U/L)**			
Mean ± SD	13.40±9.09	11.13±10.08	11.74±9.84
Median (25^th^–75^th^ percentile)	11.38 (5.30–19.0)	8.10 (4.27–14.30)	8.70 (4.40–15.0)
**LH (U/L)**			
Mean ± SD	5.02±2.34	5.46±4.02	5.36±3.72
Median (25th–75th percentile)	4.45 (3.30–6.21)	4.60 (3.20–6.75)	4.60 (3.20–6.70)
**TESTOSTERONE (ng/ml)**			
Mean ± SD	5.18±2,.94	4.49±2.08	4.64±2.30
Median (25th–75th percentile)	4.80 (3.51–6.40)	4.20 (3.40–5.20)	4,.20 (3.40–5.40)
**TESTIS VOLUME (ml)**			
Mean ± SD	11.55±4.55	12.89±4.32	12.54±4.42
Median (25th–75th percentile)	11.50 (8.0–14.0)	13.0 (9.0–14.87)	13.0 (9.0–14.50)

A) Semen phenotype of the entire study population (array-CGH and case-control study); B) Description of all analyzed patients (array-CGH and case-control study) according to their geographic origin and semen phenotype; C) Hormonal levels and testis volumes of all analyzed patients (array-CGH and case-control study).

### Methods

Germline DNA was extracted from peripheral blood samples in all the participants with standard methods.

#### Array-CGH

Customized array-CGH platforms (custom 8×60 K, Agilent Technologies, Santa Clara, CA, USA) were generated using the eArray software (http://earray.chem.agilent.com/); 53069 probes (60-mer oligonucleotides) were selected from those available in the Agilent database and cover the whole chromosome X, including Xp and Xq pseudoregions, with a medium resolution of 4 Kb. Four replicate probe groups, with every probe present in two copies on the platform, were designed in regions containing mouse infertility-associated genes i.e. sperm protein associated with the nucleus, X-linked family members (*SPANX*); testis expressed 11 *TEX11*, TAF7-like RNA polymerase II, TATA box binding protein (TBP)-associated facto *(TAF7L) and*). In these regions, the medium resolution is 2 Kb. The array also included, for the normalization of copy number changes, Agilent control clones spread along all autosomes (6842 probes). As a reference DNA, we used the same normozoospermic subject for all the study population. This control DNA was already characterized for CNV content in previous array-CGH experiments against eight different normospermic controls and presented one private gain of 27 Kb mapping to Xcentr which was not considered for the frequency analyses. 300 ng of test DNA and control DNA were double-digested with RsaI and AluI (Promega) for 1 hour at 37°C. After digestion, samples were incubated at 65°C for 20 minutes to inactivate the enzymes, and then labeled by random priming (Agilent Technologies) for 2 hours using Cy5-dUTP for the test DNA and Cy3-dUTP (Agilent Technologies) for the control DNA. Labeled DNAs were incubated at 65°C for 10 minutes and then purified with Microcon YM-30 filter units (Millipore, Billerica, USA). Every purified sample was brought to a total volume of 9.5 µl in 1xTE (pH 8.0, Promega), and yield and specific activity were determined for each sample using a NanoDrop ND-1000 UV-VIS Spectrophotometer (Labtech International LTD). The appropriate cyanine 5- and cyanine 3-labeled samples were combined in a total volume of 16 µl. After sample denaturation and pre-annealing with 5 ul of Human Cot-1 DNA (Invitrogen, Carlsbad, CA), hybridization was performed at 65°C with shaking for 24 hours. After two washing steps, the array was analyzed through the Agilent scanner and the Feature Extraction software (v10 1.1.1). Graphical overview was obtained using the DNA Analytics (v4.0.73). All the array experiments were analysed using the ADM-2 algorithm at threshold 5. Aberrant signals including 3 or more adjacent probes were considered as genomic CNVs (Figure S1). The positions of oligomeres refer to the Human Genome March 2006 assembly (hg18). All experimental data was submitted to GEO repository with the following Series accession number: GSE37948.

### Molecular genetic analyses for confirmation of array-CGH data and for the case-control study

#### Molecular analysis of deletions

For the first step screening as for the confirmatory step, we performed PCR protocol in a final volume of 10 µl containing 70 ng of genomic DNA, 3 mM MgCl2, 400 µM deoxynucleotides triphosphates, 10 pmol of specific primers, 50 U/ml of Taq DNA Polymerase (Promega PCR MASTER MIX 2X). All the primers for the first step screening had an optimal annealing temperature between 58–60°C and suspected deletions were further confirmed by i) lowering the annealing temperature (55°C); ii) performing additional PCRs with alternative primers (see details in the Table S1).

#### Molecular analysis of gains and the loss CNV31

Gains and loss CNV31 screening were performed using pre-designed TaqMan® Copy Number Assays or Custom TaqMan® Copy Number Assays (Applied Biosystems). All assays were conducted using three or four replicates for each sample (on the basis of the assay quality), in a final volume of 20 ul according to the manufacturer's instructions. The reaction mix components were: 1X TaqMan® Genotyping Master Mix, 1X TaqMan® Copy Number Assay, 1X TaqMan® Copy Number Reference Assays, 10 ng of genomic DNA. Briefly, the TaqMan® Copy Number Assay – containing two specific primers and a FAMTM dye-labeled MGB probe to detect the genomic DNA target sequence – is run in duplex with the TaqMan® Copy Number Reference Assays – containing two primers and a VIC® dye-labeled TAMRATM probe to detect the genomic DNA reference sequence. On each plate the same normozoospermic control used as reference DNA for array-CGH experiments (calibrator sample), the DNA sample of the CNV carrier and the No Template Control (NTC) were run. The CopyCaller SoftwareTM was used for post-PCR data analysis for all the copy number quantitation experiments. Information about qPCR probes are provided in Table S2.

### Statistical analysis

Statistical analyses were performed using the statistical package SPSS (version 17.0.1, Chicago, IL, USA). Non-parametric Mann-Whitney U test was performed for comparisons of: i) median values of CNV number and DNA change between patients and controls; ii) median values of sperm concentration and total sperm count in relationship with CNV number. Frequencies were compared by Fisher exact test.

## Results

### Characterization of X-chromosome linked CNVs

**Figure 1:**
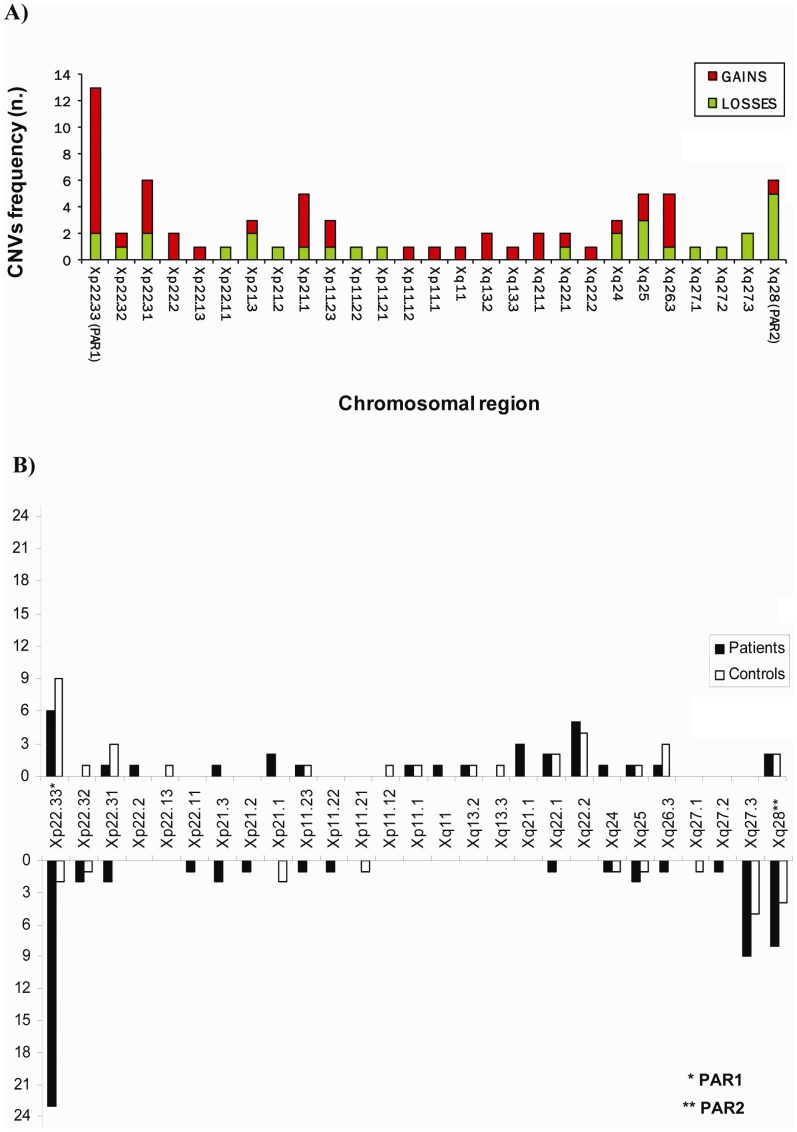
Schematic representation of the distribution of the 73 CNVs (44 gains and 29 losses) along the X chromosome identified by high resolution X chromosome specific array-CGH analysis. A) The histogram shows that the 73 CNVs were evenly distributed along the X chromosome but displayed a higher density in the pseudoautosomal region 1, PAR1 (Xp22.33). B) The frequency of gains (upwards) and losses (downwards) per X chromosome region in patients and controls are indicated.

We performed a high resolution array-CGH analysis using a microarray containing probes densely covering the complete human X chromosome (average resolution: 4 kb). Of the 199 subjects analyzed (96 idiopathic infertile subjects and 103 normozoospermic men), 97 (36 patients and 61 controls) showed the lack of CNVs, whereas the remaining 102 samples were found to carry 73 CNVs (44 gains and 29 losses) (Tables 2, 3, and 4). Thirty-two CNVs intersected genes/transcription units based on data available in genomic databases. As shown in Figure 1, CNVs were evenly distributed along the X chromosome with higher density in the PAR1.

**Table 2:** List of the 31 patient-specific (not found in normozoospermic controls) CNVs detected by array-CGH and their description according to type, gene location (*NO* = no gene found within) and occurrence in the Database of Genomic Variants (DGV).

CNV type	CNV code	Region	Size (Kb)	Start position	End position	Coding sequences within the CNV*	DGV	Frequency
**LOSSES**	**17**	Xp22.31	31.70	6,594,834	6,626,533	*NO*		1
	**18**	Xp22.31	82.00	6,756,310	6,838,310	*NO*	Variation_8908 Variation_53018 Variation_34619	1
	**22**	Xp22.11	24.35	22,969,648	22,993,997	*NO*		1
	**23**	Xp21.3	6.69	25,274,024	25,280,712	*NO*		1
	**24**	Xp21.3	67.33	26,891,769	26,959,101	*NO*		1
	**31**	Xp11.23	81.13	47,766,391	47,847,516	*ZNF630*	Variation_96640 Variation_9861 Variation_53005	1
	**32**	Xp11.22	4.15	52,065,798	52,069,943	*NO*		1
	**50**	Xq22.1	45.36	101,803,578	101,848,935	*ARMCX5-GPRASP2*		1
	**54**	Xq24	44.85	118,281,024	118,325,874	*NO*		1
	**56**	Xq25	86.07	124,632,886	124,718,959	*NO*		1
	**57**	Xq25	188.03	124,929,673	125,117,699	*NO*	Variation_52924	1
	**61**	Xq27.2	4.67	140,773,893	140,778,561	*MAGEC3*		1
	**66**	Xq27.3	7.35	145,030,566	145,037,917	*NO*		1
	**67**	Xq28	5.42	148,456,474	148,461,889	*NO*		1
	**71**	Xq28 (PAR)	290.99	154,586,913	154,877,901	*SPRY3 VAMP7*		1
**GAINS**	**1a**	Xp22.33 (PAR)	224.83	1,544	226,372	*PLCXD1 GTPBP6 PPP2R3B*		1
	**5**	Xp22.33 (PAR)	160.10	302,644	462,740	*NO*		1
	**11**	Xp22.33 (PAR)	39.73	1,347,599	1,387,328	*CSF2RA*		1
	**14**	Xp22.33 (PAR)	1.40	1,896,197	1,897,608	*NO*	Variation_31542	2
	**19**	Xp22.31	245.03	7,002,649	7,247,676	*STS HDHD1A*		1
	**20**	Xp22.2	602.10	11,104,518	11,706,614	*ARHGAP6 AMELX MSL3*		1
	**25**	Xp21.3	60.40	27,277,529	27,337,933	*NO*		1
	**26**	Xp21.1	88.57	37,168,387	37,256,960	*PRRG1*		1
	**27**	Xp21.1	9.61	37,242,364	37,251,969	*NO*		1
	**30**	Xp11.3	81.13	47,766,391	47,847,516	*ZNF630*	Variation_53003 Variation_9343 Variation_83491	1
	**38**	Xq13.2	8.98	72,202,996	72,211,976	*NO*		1
	**39**	Xq21.1	21.98	76,992,067	77,014,050	*MAGT1*		1
	**40**	Xq21.1	5.28	80,112,246	80,117,526	*NO*	Variation_83611	2
	**55**	Xq24	206.90	118,691,020	118,897,917	*SEPT6 ANKRD58 RPL39 SNORA69 UPF3B RNF113A NDUFA1*		1
	**58**	Xq25	9.68	125,143,278	125,152,957	*NO*		1
	**60**	Xq26.3	42.30	134,585,636	134,627,936	*NO*	Variation_52936	1

* CNV minimum size.

**Table 3:** List of the 33 control-specific (not found in idiopathic patients) CNVs detected by array-CGH and their description according to type, gene location (*NO* = no gene found within) and occurrence in the Database of Genomic Variants (DGV).

CNV type	CNV code	Region	Size (Kb)	Start position	End position	Coding sequences within the CNV*	DGV	Frequency
**LOSSES**	**5.B**	Xp22.33	12.63	701,071	713,696	NO		1
	**25.A**	Xp21.2	9.69	31,282,923	31,292,613	*DMD*		1
	**25.B**	Xp21.1	28.26	33,953,232	33,981,492	*NO*	Variation_7783	2
	**33.A**	Xp11.21	58.89	56,403,390	56,462,278	*NO*		1
	**53.A**	Xq24	170.73	118,278,913	118,449,646	*SLC25A43*		1
	**58.A**	Xq25	12.68	125,198,109	125,210,792	*NO*		1
	**60.A**	Xq26.3	50.84	134,801,361	134,852,198	*SAGE1*		1
	**60.D**	Xq27.1	217.83	140,175,103	140,392,930	*NO*		1
	**66.A**	Xq28	37.12	147,393,583	147,430,698	*AFF2*		1
	**71.A**	Xq28 (PAR)	122.36	154,755,542	154,877,901	*VAMP7*		1
**GAINS**	**4.A**	Xp22.33 (PAR)	237.08	153,373	390,452	*PLCXD1 GTPBP6 PPP2R3B*		1
	**5.A**	Xp22.33 (PAR)	241.98	674,222	916,206	*NO*		1
	**5.C**	Xp22.33 (PAR)	420.72	747,358	1,168,080	*NO*		1
	**12.A**	Xp22.33 (PAR)	6.61	1,693,897	1,700,511	*ASMT*		1
	**12.B**	Xp22.33 (PAR)	683.74	1,716,023	2,399,766	*ASMT DHRSX*		1
	**15.A**	Xp22.33 (PAR)	27.94	2,382,699	2,410,643	*DHRSX*	Variation_83270	1
	**15.B**	Xp22.33/22.32	280.09	4,206,493	4,486,580	*NO*		1
	**16.A**	Xp22.31	1609.42	6,487,238	8,096,662	*HDHD1 STS VCX PNPLA MIR651*		1
	**19.A**	Xp22.31	129.96	7,961,788	8,091,751	*MIR651*	Variation_9337	1
	**19.B**	Xp22.31	177.54	8,411,159	8,588,699	*KAL1*		1
	**20.A**	Xp22.2	665.88	14,590,604	15,256,487	*GLRA2 FANCB MOSPD2 ASB9 ASB11 PIGA*		1
	**20.B**	Xp22.13	13.34	18,018,894	18,032,238	*NO*		1
	**25.C**	Xp21.1	185.02	34,931,807	35,116,827	*NO*		1
	**25.D**	Xp21.1	215.00	35,269,628	35,484,626	*NO*		1
	**31.A**	Xp11.23	78.87	48,021,982	48,100,848	*SSX3*		1
	**34.A**	Xp11.12	48.06	56,870,427	56,918,489	*NO*		1
	**36.A**	Xq11	716.03	63,925,948	64,641,977	*ZC4H2 ZC3H12B*		1
	**38.A**	Xq13.2	192.04	74,375,875	74,567,915	*UPRT ZDHHC15*	Variation_74012	1
	**38.B**	Xq13.3	153.23	75,123,387	75,276,621	*NO*		1
	**55.A**	Xq25	53.80	120,385,787	120,439,584	*NO*		1
	**59.A**	Xq26.3	24.69	134,151,039	134,175,725	*NO*		1
	**60.B**	Xq26.3	13.45	136,050,422	136,063,872	*NO*		1
	**60.C**	Xq26.3	91.26	137,089,527	137,180,783	*NO*		1

* CNV minimum size.

**Table 4:** List of CNVs found by array-CGH considering their occurrence in controls and in patients with their description according to type, gene location (NO = no gene found within) and presence in the Database of Genomic Variants (DGV).

	CNV code	CNV type	Region	Size (Kb)	Start position	End position	Coding sequences within the CNV*	DGV	Frequency in patients	Frequency in controls
**Control-enriched CNVs**	12	GAIN	Xp22.33 (PAR)	17.30	1,693,897	1,711,194	*ASMT*	Variation_83259	1	3
**Patient-enriched CNVs**	15	LOSS	Xp22.33 (PAR)	1.40	1,896,197	1,897,608	*NO*	Variation_104545	23	1
	64	LOSS	Xq27.3	3.92	143,436,347	143,440,268	*NO*	Variation_115340	8	5
	69	LOSS	Xq28	11.77	154,044,877	154,056,645	*NO*		7	3
**Common CNVs**	16	LOSS	Xp22.32	7.76	4,250,413	4,258,174	*NO*	Variation_52995	2	1
	35	GAIN	Xp11.1	117.14	57,318,438	57,435,573	*FAAH2*		1	1
	49	GAIN	Xq22.1	5.30	100,942,190	100,947,490	*NO*		2	2
	51	GAIN	Xq22.2	34.69	103,152,319	103,187,013	*H2BFWT H2BFM*	Variation_3254	5	4
	68	GAIN	Xq28	105.66	148,686,631	148,792,286	*MAGEA8*	Variation_31571	2	2

* CNV minimum size.

Since homologous sequences at the border of a CNV may act as a substrate for non-allelic homologous recombination (NAHR), we checked the nature of regions flanking (between the minimum-maximum size of the CNV and approximately up to 1 Mb from the maximum size) the identified CNVs in order to understand whether NAHR is likely to occur (UCSC Genome Browser). Highly homologous sequences were identified only in 19% of CNVs, indicating that NAHR is not involved in the majority of observed CNVs. This figure was concordant with other observations reporting a similar frequency of potential NAHR targets [15]. It is interesting to note that in some areas (Xp11.12-q21.1) only duplications were found, whereas from Xq27.1-q27.3 only deletions were detected. One of the PAR1-linked losses (CNV15) was found in 23 patients and only once in controls (Figure 1b). This small CNV has already been described in the Database of Genomic Variants (DGV) both as loss and gain. This CNV was situated inside a 3914 bp Simple Tandem Repeat which included two Segmental Duplications (respectively of 1498 bp and 1444 bp) that therefore may act as substrate for NAHR. This mechanism may have lead also to reciprocal duplication and in fact CNV14, identified in our study, is the reciprocal duplication of CNV15. No genes were identified inside or nearby CNV14/15 which made it difficult to attribute a pathogenic role to this loss. Moreover, the same sequence was present also on the Y chromosome which further complicated the interpretation of the results.

**Figure 2:**
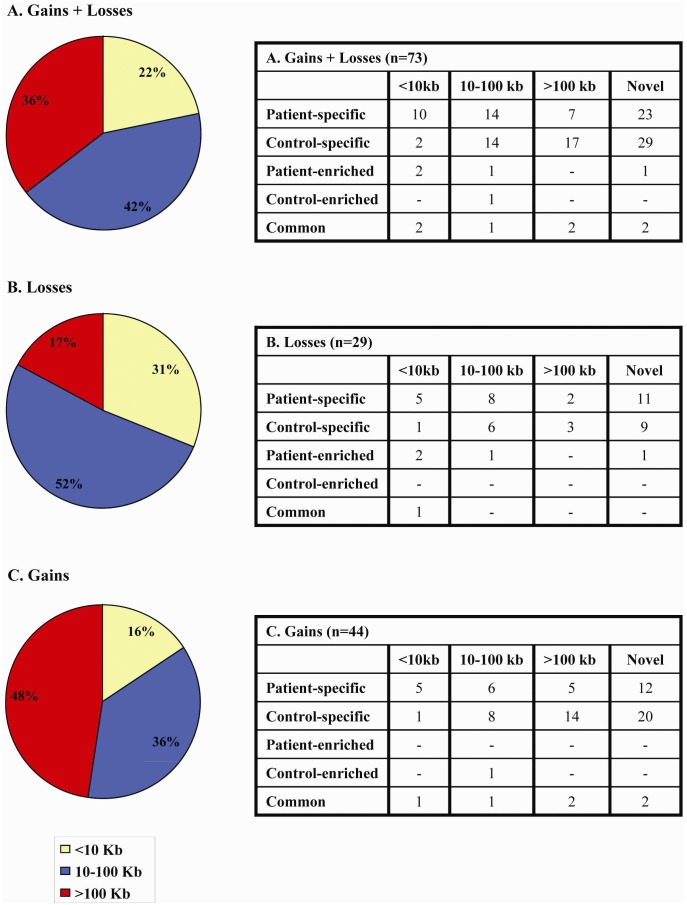
Array-CGH study. : distribution of the 73 CNVs according to their size: small (<10 Kb), medium (10–100 Kb) and large (>100 Kb) referred to A) all CNVs (44 gains and 29 losses); B) losses; C) gains. Losses were typically of small/medium size (52%) whereas gains are generally of larger size (48%). On the side, tables display the number of A) all CNVs; B) losses; C) gains of different size and categorized according to their occurrence in patients/controls: i) “patient-specific” when found only in patients; ii) “control-specific” when found only in controls; iii) “patient-enriched” when found predominantly in patients; iv) “control- enriched” when found predominantly in controls; v) “common” when found at a similar frequency in patients and controls.

Considering the size of detected CNVs, which ranged from 1.4 Kb to 1609 Kb (Tables 2, 3, and 4), we noticed that losses were typically of small/medium size and only 17% of them were large (Figure 2). Conversely, large gains represented 48% of the total CNVs and the difference between frequencies of losses and gains of >100 Kb was statistically significant (p = 0,012). Small CNVs (<10 Kb) were more frequently found in patients in respect to controls whereas large gains have been found mainly in controls (Figure 2).

According to the Database of Genomic Variants (DGV) website, losses/gains were divided into “known” and “novel”, identifying 21 novel losses and 34 novel gains (Tables 2, 3, and 4). Among the 73 CNVs, 31 (15 losses and 16 gains) were found only in patients, “patient-specific” (Table 2) and 33 (10 losses and 23 gains) were found only in the control group, “control-specific” (Table 3). Of the remaining 9 CNVs, only one gain (CNV12) was found more frequently among controls whereas those resulting more frequent among patients (“patient-enriched”) were deletions. The rest (4 gains and 1 deletion) were found to equally occur in both patients and controls (Table 4). These data suggest that gains are less likely to affect spermatogenesis since 63% of them (28/44) were found also in normozoospermic controls. On the contrary, deletions were less frequent in controls (11/29; 38%) indicating that in the presence of a deletion an abnormal sperm phenotype is more likely to occur. A general outline of the array-CGH findings with phenotypic description is provided in Table S3.

### CNV burden

In order to assess the potential impact of CNVs in cases versus controls, we used two primary measures of CNV burden: the mean size and the mean number of CNVs/individual (Table 5A). The mean value of losses bp was significantly higher in patients than in controls (11.79 Kb and 8.13 Kb, respectively; p = 3.435×10^−4^). All losses were confirmed by PCR plus/minus or Real Time PCR, except for PAR-linked losses (n = 4), for which no suitable assay could be designed. The number of CNVs/person was significantly higher in patients compared to controls (p = 0.002) and depended on the overrepresentation of losses in the former group (0.57 versus 0.21; p = 8.785×10^−6^) (Table 5). CNV15, the most frequently found loss appears to be the major contributor to the deletion burden, however even without this loss the number of losses/person is significantly higher in the patient's group (p = 0.041). Phenotypic description of patients (loss-carriers and no CNV-carriers) is provided in Table S4. Although the frequency of patients with more than one CNV (n = 19; 19.8%) was nearly twice that of controls (n = 11; 10.7%), the difference did not reach statistical significance (p = 0.078). On the other hand, comparing the frequencies of subjects with ≥1 CNV in cases versus controls, we observed a highly significant difference when considering the total number of CNVs (p = 0.003) and of losses (p<0.001) (Table 5B).

**Table 5:** Array-CGH study: Comparison between patients and controls of the mean number and mean extension of CNVs (A) as well as the number of all subjects bearing more than one CNV (B).

A	PATIENTS (n = 96)		CONTROLS (n = 103)		p	
	Mean CNV number ± sd	Mean CNV extension (Kb) ± sd	Mean CNV number ± sd	Mean CNV extension (Kb) ± sd	p1	p2
**LOSSES+GAINS**	0.87±0.85	36.21±85.4	0.54±0.76	73.87±222.08	2.095×10^−3^	0.113
**LOSSES**	0.57±0.64	11.79±38.43	0.21±0.46	8.13±32.30	8.785×10^6^	3.435×10^−4^
**GAINS**	0.30±0.54	24.42±76.50	0.33±0.62	65.74±220.07	0.862	0.733

B	PATIENTS (n = 96)	CONTROLS (n = 103)	p	OR (95% CI)
**≥1 CNV/subject**	60	42	0.003	1.5 (1.2–2.0)
**≥1 loss/subject**	47	20	<0.001	2.5 (1.6–3.9)
**≥1 gain/subject**	25	28	0.874	-

sd = standard deviation. OR = odds ratio. CI = confidence interval. *p*1 refers to the mean number of CNV/subject. *p*2 refers to the mean DNA change/subject.

### CNVs and semen parameters

A significant association with sperm concentration and total sperm number was observed among patients when considering the total CNV number (Table 6). Patients with more than 1 CNV had a significantly lower sperm concentration and total sperm count than those with ≤1 CNV (0.2±0.6×10^6^/ml versus 1.0±2.0×10^6^/ml; p<0.022; 2.3±4.6×10^6^ versus 1.0±3.3×10^6^; p<0.032). The maximum number of CNVs/subject was three, and of the five patients with three CNVs four were azoospermic and one was severely oligozoospermic with <1 million spermatozoa/ejaculate (Table S6). All of them had at least one private CNV (uniquely found in this patient), and only one patient (07-170) shared two recurrent CNVs with two others (07-13, 07-30). Given that the selection of patients was based on the absence of known causes of spermatogenetic failure, subjects with multiple CNVs did not show any additional andrological anomaly or other relevant diseases. Semen parameters and testis histology of patients and controls with >1 CNVs are reported in Table S5, 6.

**Table 6:** Array-CGH study: comparison of patients' semen parameters according to the number of CNVs.

PATIENTS (n = 96)				
	SPERM CONCENTRATION (n×10^6^/ml)	p	TOTAL SPERM NUMBER (n×10^6^)	p
**0 CNV (n = 36)**	1.2±2.4 (0.01; 0.0–12.0)		2.9±5.7 (0.01; 0.0–30)	
**≥1 CNV (n = 60)**	0.6±1.3 (0.0; 0.0–6.2)	0.068	1.6±3.4 (0.0; 0.0–17.4)	0.075
**0 LOSS (n = 49)**	1.0±2.1 (0.01; 0.0–12.0)		2.7±5.0 (0.01; 0.0–30.0)	
**≥1 LOSS (n = 47)**	0.6±1.5 (0.0; 0.0–6.2)	0.053	1.4±3.6 (0.0; 0.0–17.4)	0.051
**0 GAIN (n = 71)**	1.0±2.1 (0.0; 0.0–12.0)		2.4±4.9 (0.0; 0.0–30.0)	
**≥1 GAIN (n = 25)**	0.4±0.7 (0.0; 0.0–2.3)	0.185	1.3±2.3 (0.0; 0.0–6.4)	0.215
**≤1 CNV (n = 77)**	1.0±2.0 (0.0; 0.0–12.0)		2.3±4.6 (0.0; 0.0–30.0)	
**>1 CNV (n = 19)**	0.2±0.6 (0.0; 0.0–2.0)	0.022	1.0±3.3 (0.0; 0.0–13.4)	0.032
**≤1 LOSS (n = 88)**	0.9±1.9 (0.0; 0.0–12.0)		2.1±4.4 (0.0; 0.0–30.0)	
**>1 LOSS (n = 8)**	0.2±0.6 (0.0; 0.0–1.8)	0.230	1.7±4.7 (0.0; 0.0–13.4)	0.309
**≤1 GAIN (n = 92)**	0.8±1.9 (0.0; 0.0–12.0)		2.2±4.5 (0.0; 0.0–30.0)	
**>1 GAIN (n = 4)**	0.0±0.0 (0.0; 0.0–0.01)	0.293	0.0±0.0 (0.0; 0.0–0.01)	0.29

Sperm concentration and total sperm number are expressed as: mean ± standard deviation (median; range). Significance is depicted by a *p* value<0.05.

### Screening for selected deletions

To further investigate the potential clinical implications of losses, 13 patient-specific deletions were subsequently screened in a large group of infertile and normozoospermic men: excluding CNV66, they all remained patient-specific (Table 7). Due to the rarity of the 12 patient-specific losses, statistically significant differences were not observed in their frequencies compared to the control group. In fact, 8/12 were private (found in a single individual) whereas only 4 were recurrent with a still relatively low frequency (0.5–1.1%).

**Table 7:** Case-control study of selected losses preliminarily identified by array-CGH as patient-specific (not found in normozoospermic controls).

CNV code	X chr. band	Patients (frequency)	Controls (frequency)	p value	Carriers code	Carriers phenotype (total sperm count ×10∧^6^ and/or testis histology)	Genes inside and nearby (<500 Kb)
17	Xp22.31	2/359 (0.55%)	0/370 (0%)	0.244	A448, A828	A448: Oligozoosp. (13.4); A828: Oligozoosp. (20)	*NLGN4X*, *VCX3A* §, *HDHD1A* †, *STS*
18	Xp22.31	1/359 (0.27%)	0/370 (0%)	0.492	07-96	Azoosp. (0.0; SCOS)	*VCX3A* §, *HDHD1A* †, *STS*
22	Xp22.11	1/359 (0.27%)	0/370 (0%)	0.492	MMP718	Oligozoosp. (6.4)	*DDX53* §, *PTCHD1* †, *PRDX4*
23	Xp21.3	1/359 (0.27%)	0/370(0%)	0.492	A142	Cryptozoosp. (0.01)	*POLA1*, *SCARN23*, *ARX* §
31	Xp11.23	2/270 (0.74%)	0/325(0%)	0.206	A630, 09-126	A630: Oligozoosp. (7.2) 09-126: Oligozoosp. (8)	*ARAF, SYN1, TIMP1, CFP, ELK1, UXT, LOC100133957, CXXC1P1, ZNF81, ZNF182* §, *SPACA5* *, ***ZNF630,*** * SPACA5B* *, *LOC100509575* *, *SSX5* †, *SSX1, SSX3, SSX4, SSX4B, SLC38A5,FTSJ1,PORCN,EBP, TBC1D25, RBM3, WDR13*
32	Xp11.22	2/359 (0.55%)	0/370(0%)	0.244	A162, 08-190	A162: Cryptozoosp. (0.01); 08-190 Azoosp (0.0)	*MAGED1, SNORA11D, SNORA11E, MAGED4B/MAGED4* §, *XAGE2* †, *XAGE2B, XAGE1B, XAGE1A, XAGE1D, XAGE1C, XAGE1E*
50	Xq22.1	1/359 (0.27%)	0/370 (0%)	0.492	07-22	Azoosp. (SCOS)	*NXF2B, NXF2, TMSB15A, NXF4 ARMCX5, GPRASP1* §, ***ARMCX5-GPRASP2*** *, GPRASP2* †, *BHLHB9, RAB40AL, BEX1, NXF3*
54	Xq24	1/359 (0.27%)	0/370 (0%)	0.492	MM550	Oligozoosp. (0.24)	*LONRF3, KIAA1210, PGRMC1* §, *SLC25A43* †, *LOC100303728, SLC25A5, CXorf56, UBE2A, NKRF, SEPT6, MIR766*
56	Xq25	1/359 (0.27%)	0/370 (0%)	0.492	06-188	Azoosp. (0.0; SCOS)	*LOC10012952O, DCAF12L2* §
57	Xq25	1/359 (0.27%)	0/370(0%)	0.492	05-238	Cryptzoosp. (0.22)	*DCAF12L2* †, *DCA12L1*
61	Xq27.2	1/359 (0.27%)	0/370 (0%)	0.492	07-30	Azoosp. (0.0; SCOS)	*SPANXA2,SPANXA1,SPANXD,SPANXE* §, ***MAGEC3*** *, MAGEC1* †, *MAGEC2*
66	Xq27.3	1/359 (0.27%)	1/370 (0.2%)	1.000	07-516, CS67	07-516: Azoosp. (0.0; mixed SCOS-hypospermatogenesis); CS67: Normozoosp. (235)	*CXorf1, MIR890, MIR888, MIR892A, MIR892B, MIR891B, MIR891A* §
67	Xq28	4/359 (1.11%)	0/370 (0%)	0.058	05-196, MMP676, MMP687, MMP704	05-196: Azoosp. (0.0; SCOS); MMP676: Oligozoosp. (21.5); MMP687: Oligozoosp.(57.2); MMP704: Oligozoosp (1.02).	*IDS, LOC100131434, CXorf40A* §, *MAGEA9B* *, *HSFX2* †, *HSFX1* †, *TMEM185A, MAGEA11, HSFX1, HSFX2, MAGEA9, MAGEA8, CXorf40B*

Genes inside the CNV minimum size are depicted in bold;

* genes inside the CNV maximum size;

§ the first proximal flanking gene;

† the first distal flanking gene; the remaining genes are situated <500 Kb from the minimum size border. Azoosp = Azoospermia; Oligozoosp = Oligozoospermia; Cryptozoosp = Cryptozoospermia; SCOS = Sertoli Cell Only Syndrome; SGA = Spermatogenic Arrest.

#### Recurrent patient-specific CNVs

Among the patient-specific recurrent CNVs, three deletions are of major interest. CNV67, observed in 1.1% of patients may remove (considering its maximum size) the melanoma antigen family A, 9B (*MAGEA9B*), which belongs to the Cancer Testis Antigens (CTAs) gene family, expressed exclusively in the testis with the highest expression level in spermatocytes and in some tumour cell lines [16]. This deletion may also affect additional genes with prevalent or exclusive expression in the testis such as other CTAs and the following: transmembrane protein 185A (*TMEM185A*), chromosome X open reading frame 40A (*CXorf40A*), X linked heat shock transcription factor family (*HSFX*) all situated at <1 Mb from the deletion. Phenotypes of patients with this deletion ranged from azoospermia due to Sertoli Cell Only Syndrome (SCOS, [17]) to oligozoospermia. CNV 31 presents a reciprocal duplication (CNV30, Table 2) and was observed in 4 patients (two found by array-GH and two by qPCR) and 0/325 controls. CNVs 30/31 affect the dosage of zinc finger protein 630 (*ZNF630*), a gene with unknown function; however, considering their maximum extension, additional genes with exclusive expression in the testis such as the sperm acrosome associated 5 *SPACA5*,/*SPACA5b*) are also involved. CNV32 does not remove any gene directly, but it is situated within an area abundant in CTA genes. In order to define whether the underlying mechanism of these deletions is NAHR we analyzed the flanking regions. Only CNV 30/31 showed Segmental Duplications (SD) which may explain the recurrence of deletion/duplication events. Although also CNV67 was found in 4 patients, this deletion does not have a reciprocal duplication and it is not flanked by SDs. An alternative mechanism for the formation of CNV67 could be Non Homologous End Joining (NHEJ), since substrates for this mechanism are highly represented in this area (many LINE and Alu elements). However this hypothesis requires further confirmation by the fine mapping of the breakpoints.

#### Private patient-specific CNVs

Concerning private patient-specific deletions, which were found only in single patients, we observed two deletions directly affecting gene dosage. CNV50 removes the ARMCX5-GPRASP2 read-through (*ARMCX5-GPRASP2*) genes for which no testis expression data are available. The carrier of this deletion suffers from azoospermia due to SCOS. CNV61, observed in one azoospermic man, removes another CTA family member, the melanoma antigen family C, 3 *MAGEC3*. This deletion may also affect other neighbouring CTA genes, such as the melanoma antigen family C, 1 *MAGEC1* and Sperm protein associated with the nucleus, X-linked, family member E (*SPANXE*). Four deletions (CNV22, 54, 56 and 57) contained several (from 4–32) conserved transcription factor binding sites, but the neighbouring genes were relatively distant (from 8 Kb to 400 Kb).

## Discussion

The diffusion of assisted reproductive techniques as a therapeutic option in severe male factor infertility raised several questions about the short and long-term consequences on the offspring, since infertile men are at higher risk of being carriers of genetic anomalies in both their genomic DNA and gametes. Although the importance of diagnosing genetic factors in this category of future fathers is fully recognized, the diagnostic workup of infertile men is still limited to a few genetic tests. Our working hypothesis was that, similarly to Y chromosome-linked CNVs (AZF and gr/gr deletions), we would be able to identify recurrent, pathogenic deletions on the X chromosome. First, an X-chromosome specific high resolution array-CGH analysis was carried out in 199 men with known sperm count and was followed by a screening of selected CNVs in several hundred infertile patients and normozoospermic controls. Our array-CGH analysis showed that 50% of subjects presented at least one CNV, and the majority of these CNVs (55/73) were not reported in currently available databases of genomic variants. Among the few X-linked CNVs reported in subjects with known sperm count [7] only six partially or completely overlapping CNVs were found. This can be due to both technical issues (different array resolution, different criteria used for the interpretation of data, lack of validation in the Tuttelmann paper) and/or due to the patient selection criteria (azoospermic men were selected for a specific histology, called SCOS, in the Tuttelmann et al paper [7]). Interestingly, a small deletion, CNV 69 on Xq28 was observed in 7 patients and 3 controls and it maps inside a CNV reported by Tuttelmann et al [7] as patient-specific, present in a single oligozoospermic German man (“private”). This discrepancy is likely due to the larger size (34 Kb) of the patient-specific deletion in the German patient compared to our 10 subjects (11.7 kb). On the contrary, a reciprocal deletion/duplication (CNV31/CNV30) was observed exclusively in patients (n = 4) in our study, whereas Tuttelmann et al. found two normozoospermic carriers of the duplication and one carrying the deletion [7]. However, the deletion encountered in the above German study was 25 Kb smaller than CNV31/30. An other interesting finding concerns two partially overlapping gains detected in both studies, which affect the dosage of two genes (*H2BFWT* and *H2BFM*). In our study this CNV (CN51) has been found both in controls (n = 4) and patients (n = 5), whereas in the German study [7] it was found only in an oligozoospermic patient. Given that the larger CNV reported in the German study [7] duplicates also two other genes (*TMSB15B*, *H2BFXP*), the combined analysis of the results suggests that it is more likely that the not shared genes, situated in the larger duplication, are responsible for the observed oligozoospermic phenotype.

The further analysis of patient-specific deletions (n = 13) revealed that >90% of them are unique or rare (frequency <1%). These data are in line with the previous whole genome array-CGH study [7] in which among the 27 patient-specific CNVs only one recurrent duplication was found in two oligozoospermic men. Similarly in the paper by Stouffs et al, among the 10 patient specific autosomal CNVs only two were recurrent [8]. The role of rare CNVs has already been established for other multifactorial diseases [18], [19] and since mutations causing spermatogenic failure are unlikely transmitted to the next generation, we can predict that *de novo* mutations probably play a major role in primary testicular failure. It remains difficult to ascertain the importance of rare patient-specific CNVs in spermatogenesis through family analysis, since analysis on maternal X-chromosome would not be informative and brothers (with a 50% chance of sharing the same X chromosome) were not available for analysis. The difficulty to obtain DNA from relatives in relationship with infertility studies is related to the delicate nature of this condition and for this reason the two previous array-CGH studies were also unable to define the *de novo* nature of the identified CNVs. As an alternative way to explore their potential clinical relevance, we performed a search for functional genomic regions (protein coding genes, microRNAs, conserved transcription binding sites) mapping inside or nearby the 13 deletions of interest. Since men are hemizygous for X-linked genes, their CNV-dependent altered expression cannot be compensated by a normal allele and could potentially lead to a direct pathological effect. Ours is the first study suggesting that X-linked CTA family members are recurrently affected and their dosage variation may play a role in CNV-related spermatogenic failure. CTA genes comprise more than 240 members from 70 families and are generally divided into two broad categories: X-linked (mostly multicopy genes) and non-X CTA genes (mainly single copy genes located on autosomes) [for review see [16], [20]]. These genes are normally expressed only in germ cells but aberrant activation has also been reported in a number of malignant tumors. The exclusive physiological expression in germ cells strongly suggests a role in spermatogenesis hence human CTA gene family members are largely unexplored and no clinical data is available. Interestingly, by tracing the evolutionary history of CTA genes, it has been demonstrated that CTA genes in general and the X chromosome linked CTA genes in particular are under strong diversifying pressure and amongst the fastest-evolving genes in the human genome [21]. Consequently, many of the human X-linked CTA genes do not have easily identifiable orthologues in the mouse or rat genomes, which makes it difficult to study the role of these genes in animal models. Clues regarding functionality of CTAs for many of these proteins point to a role in cell cycle regulation or transcriptional control [for review see [22]]. Data obtained in the 103 controls (array-CGH analysis) indicates that in this group only one control-specific deletion contained a CTA gene, the sarcoma antigen 1 *SAGE1*, which indicates that this gene is unlikely a spermatogenesis candidate gene. In support of such a statement, the expression of this gene is extremely low in the testis. On the contrary, for the patient-related CTA genes expression levels in the testis and germ cells were substantially higher. Apart from CTA family members we identified other potential candidate genes in the patient group which deserve further genetic screening. On the contrary, we can conclude that those genes which are deleted in control subjects, are unlikely to be spermatogenesis candidate genes since their absence is compatible with normal spermatogenesis. Among the 6 gene-containing control–specific losses, with the exception of vesicle-associated membrane protein 7 (*VAMP7*), the level of testicular expression is either absent or very low. *VAMP7* is situated in PAR2 and it has been described as strongly expressed in the testis, especially in spermatids. Our data indicates that *VAMP7* haploinsufficiency (i.e. one copy of the gene is still retained on the Y-linked PAR2) does not impair spermatogenesis.

One of the most stimulating findings of our article is related to the CNV burden observed in the patients' group in relationship with loss of genetic material. The relatively high frequency of Y chromosome deletions (4–7% in severe spermatogenic failure) already suggested that infertile men are more prone to the loss of genetic material [11]. The mechanism by which Y chromosome deletions lead to spermatogenetic failure is not fully clarified and they may act either by removing genes involved in spermatogenesis or by affecting meiosis. Here we found an excess of X-linked CNV number and DNA loss in patients with reduced sperm count, which was only partially related to direct gene removal, hence the majority of deletions mapped close to gene-rich areas. We also found a significant association between CNV number and sperm count in the infertile group, which further reinforces the potential link between deletion burden and spermatogenic failure. Similarly to our data, in the paper by Tuttelmann et al [7] a significant inverse correlation has been found between sperm count and CNV number at the whole genome level.

Whether the observed deletions are directly responsible for the phenotype (either affecting gene expression or interfering with sex chromosome pairing for those mapping to the PAR regions) or simply arise due to increased genomic instability, remains a puzzling question. Some previous observations suggest a possible relationship between genomic instability and male infertility and are related to microsatellite instability [23] as well as to the presence of multiple CNVs on the Y chromosome in men with AZF deletions [24] and an excessive CNV number in azoospermic men with SCOS [7]. Previously, we also observed a significant effect of multiple rearrangements in the AZFc region on sperm production, suggesting a potential link between a less stable genome and spermatogenic efficiency [25]. Additionally, epidemiological observations showing a higher incidence of morbidity (including cancer) and lower life expectancy [22], [26] in infertile men would support a potential link between altered spermatogenic function and genomic instability. Our study suggests a potential involvement of increased X-linked deletion burden in the aetiology of impaired spermatogenesis and stimulates further research to better define its implication in primary testicular failure and on general health issues for both the patient and his future offspring.

In conclusion, by the analysis of the X chromosome, at the highest resolution available to date, in a large group of subjects with known sperm count we were able to provide evidence about the lack of highly recurrent deletions, which suggest that an AZFc-like region does not exist on this sex chromosome. Our investigation gives an important contribution both to the field of genetics and reproductive medicine since we identified a large number of novel CNVs, and by our second step analysis, we confirmed 12 deletions as being specific to men with impaired spermatogenesis. The analysis of gene-containing CNVs in patients and in controls allows to discern between those that merit future research and those which are unlikely to be involved in spermatogenesis.

## Supporting Information

Figure S1 **Array-CGH profiles of two CNVs detected by customed oligonucleotide-based X microarray.** Magnified view of CNV 30 (left) and CNV 50 (right) in cases 08-79 and 07-22, respectively. The shaded areas indicate a gain in DNA copy number (duplication, average log2 ratios: +1) detected by red dots (left) and a deletion (average log2 ratios: −4) detected by green dots (right). Arrows indicate the first and the last oligonucleotide duplicated (left) or deleted (right), respectively.
(TIF)

Table S1 **List of primers used for the validation of array-CGH results and for the case-control study.**
(DOC)

Table S2 **List of TaqMan Copy number assay codes used for the validation process.**
(DOC)

Table S3 **A general outline of the array-CGH findings with phenotypic description of patients and controls.**
(XLS)

Table S4 **Phenotypic features according to the presence/absence of losses in patients, including the comparison between carriers and no-CNV carriers of hormonal parameters and testis volumes (A) as well as the description of patients with losses detected during both the array-CGH and case-control studies (B).**
(DOC)

Table S5 **Array-CGH study: comparison of semen parameters according to the number of CNVs in the control group.**
(DOC)

Table S6 **Array-CGH study: Spermatogenic characteristics of patients and controls carrying more than one CNV.**
(DOC)
