# Supplementary figures and images for: High Resolution X Chromosome-Specific Array-CGH Detects New CNVs in Infertile Males
Source: PLoS One. 2012 Oct 9;7(10):e44887. doi: 10.1371/journal.pone.0044887 (PMC3467283; doi:10.1371/journal.pone.0044887)

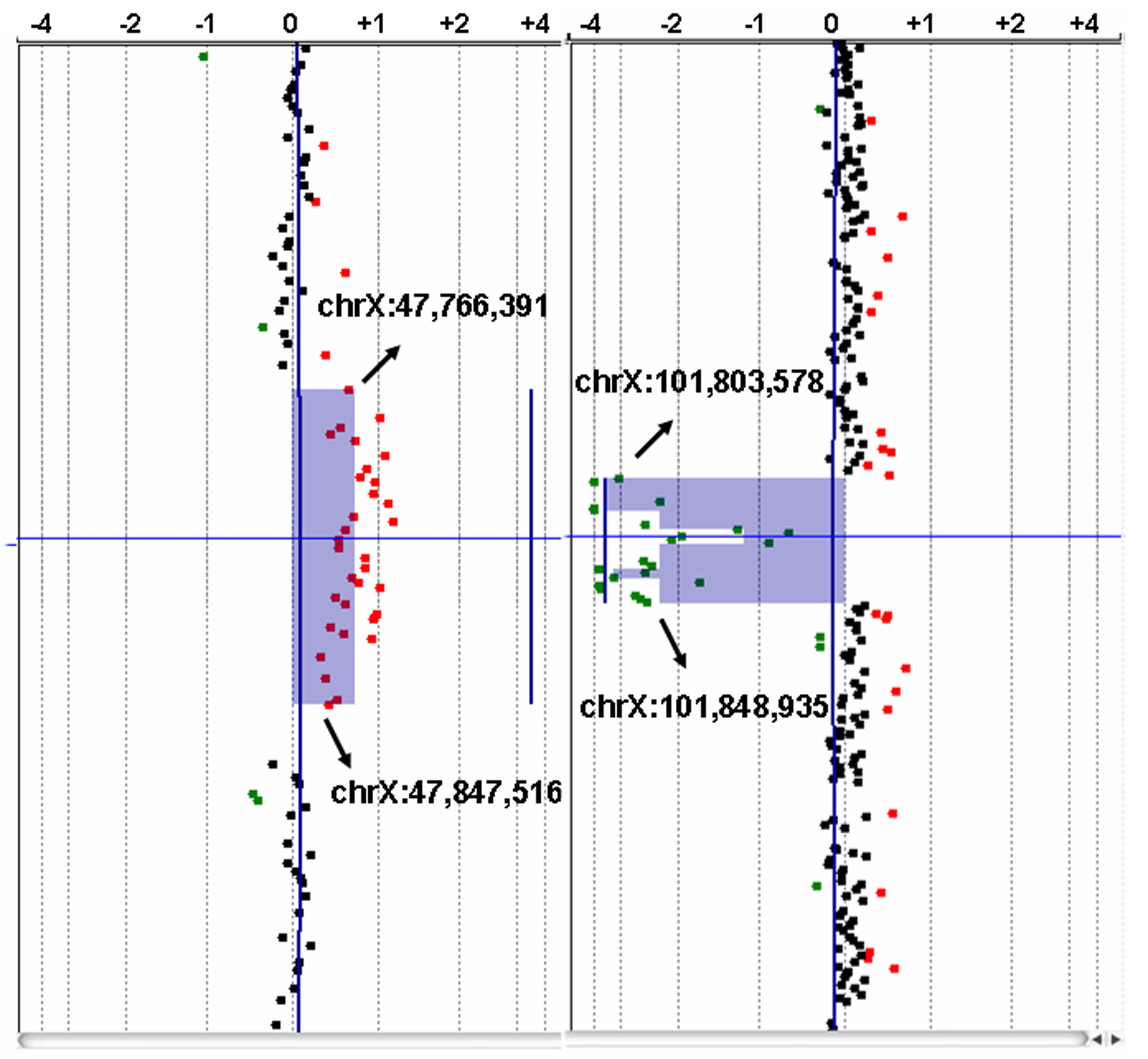

Supplement: Figure S1 — Array-CGH profiles of two CNVs detected by customed oligonucleotide-based X microarray. Magnified view of CNV 30 (left) and CNV 50 (right) in cases 08-79 and 07-22, respectively. The shaded areas indicate a gain in DNA copy number (duplication, average log2 ratios: +1) detected by red dots (left) and a deletion (average log2 ratios: −4) detected by green dots (right). Arrows indicate the first and the last oligonucleotide duplicated (left) or deleted (right), respectively. (TIF) [file pone.0044887.s001.tif]
