# Supplementary material for: High Resolution X Chromosome-Specific Array-CGH Detects New CNVs in Infertile Males
Source: PLoS One. 2012 Oct 9;7(10):e44887. doi: 10.1371/journal.pone.0044887 (PMC3467283; doi:10.1371/journal.pone.0044887)
Supplement: Table S1 — List of primers used for the validation of array-CGH results and for the case-control study. (DOC) [file pone.0044887.s002.doc]

| **LOSS code** | **PRIMERS FOR FIRST STEP SCREENING** (5’→ 3’) Forward. Reverse | | **PRIMERS FOR CONFIRMATION** (5’→ 3’) Forward. Reverse | |
| --- | --- | --- | --- | --- |
| 16 | **del Xp22.32-F1** | **TTATTGGTGGCGGGGTATTA** | del Xp22.32-F2 | TCTTTGCTCTCCTCGCAAAT |
|  | **del Xp22.32-R1** | **TATGTTTGGCAGGCATTTGA** | del Xp22.32-R2 | GCGAATTTGGTGAATGTGTG |
| 17 | **del Xp22.31B-F4** | **GTCCTGCCTTTGACCACATT** | del Xp22.31B-F3 | TTCATGGGAAACAACTGCAA |
|  | **del Xp22.31B-R4** | **CGTGGACAGGGTTCTTCATT** | del Xp22.31B-R3 | TGACAAATGCAAGGTGGAAA |
| 18 | **del Xp22.31-F1** | **CTGGTGTTAGGCCGTGAAAT** | del Xp22.31-F2 | GCTCCCAATGGATTTGAGAA |
|  |  |  | del Xp22.31-R2 | AGGCTAAAAGAGGCCCAGAG |
|  | **del Xp22.31-R1** | **GCATGAACCTGAACAGAGCA** | del Xp22.31-F3 | CAACAGGAAGCGAGTTGTCA |
|  |  |  | del Xp22.31-R3 | CCTGGTGGTGGAGACAGTTT |
| 22 | **del Xp22.11-F1** | **ACTTTTCCCAGCTTTGCTCA** | del Xp22.11-F2 | CCCCACGTATTGGTTAATGG |
|  |  |  | del Xp22.11-R2 | TGAGATGCATTCCATTCCAA |
|  | **del Xp22.11-R1** | **GGCAGGAGCTAAGAATGCAC** | del Xp22.11-F3 | ACACACACACCCTGACTCCA |
|  |  |  | del Xp22.11-R3 | CGTGTTTGGAACCTCCATCT |
| 23 | **del Xp21.3-F1** | **ATTTTGGTTCCTCTGCATGG** | del Xp21.3-F2 | AAGCCCACACCACATTCTTC |
|  |  |  | del Xp21.3-R2 | TGACCTGGGAGCAGTTCTCT |
|  | **del Xp21.3-R1** | **CCCTGGTAGCCACCATTCTA** | del Xp21.3-F3 | CTCTGAGTCACCCACCGTCT |
|  |  |  | del Xp21.3-R3 | ACCCCTGTTAAAGGGCTGAT |
| 24 | **Xp21.3b-F3** | **AAGGGAGGGAGGAAGTTTGA** | delXp21.3b-F1 | CACGCTGAAGTCATCCAGAA |
|  | **Xp21.3b-R3** | **GCTGGAGCAGATCACAACAA** | delXp21.3b-R1 | CAAAAGCAATGGGGTTCACT |
| 25.A | **delXp21.2-F1** | **TGCTGAAGTCTGCAACAACC** |  |  |
|  | **delXp21.2-R1** | **GTCACTTGCCAGAGGAGGAG** |  |  |
| 25.B | **delXp21.1-F1** | **CCTGTGGAGCTGTGAGTCAA** |  |  |
|  | **delXp21.1-R1** | **AAATGCCAGTAGCACCCAAC** |  |  |
| 32 | **delXp11.22B-F2** | **GAAATTGTGGCACACCACTG** | delXp11.22B-F3 | TCCTGATGGATCTTTTGACCA |
|  | **delXp11.22B-R2** | **GGGGATCCATAGAACAGCAA** | delXp11.22B-R3 | CTCAGTGGTGCCTTGGAGAT |
| 33.A | **delXp11.21-F1** | **CGTTTCATGGTTTCCGAGTT** |  |  |
|  | **delXp11.21-R1** | **CCCATAAAATCCCCAGACCT** |  |  |
| 50 | **del Xq22.1-F1** | **GGTGCTTGTGTTTGCTGATG** | del Xq22.1-F2 | ACTGGAGCCTTATCCCACCT |
|  | **del Xq22.1-R1** | **TTGTTTGAGCCAGGGATTCT** | del Xq22.1-R2 | TTTGTGAGCTTTTGCCCATT |
| 53.A | **delXq24-F1** | **CGTGCGTATGTGTTTCATCC** |  |  |
|  | **delXq24-R1** | **CCACTGGCCCACATCTTAGT** |  |  |
| 54 | **del Xq24-F1** | **CTTCTGCACGTTTGAGGACA** | del Xq24-F2 | TGCTGGAACAATGACCTTGA |
|  |  |  | del Xq24-R2 | GACACTTTCGGTTTGCCCTA |
|  | **del Xq24-R1** | **TCCTCTGTGCTGAAGGGTCT** | del Xq24-F3 | AGTGAACAGGGAGAGGCAGA |
|  |  |  | del Xq24-R3 | CCCAAAAGCTGAAGTCCAAG |
| 56 | **delXq25B-F2** | **ATTGCAGTCAGAGCCTTGGT** | delXq25B-F3 | TGGGTGGTGGTATGACATTG |
|  |  |  | delXq25B-R3 | TTGGCTCCCAAATAAGATGG |
|  | **delXq25B-R2** | **GCAGATTGGCTGGTCATCTT** | delXq25B-F4 | TATTTCCATGGACAGCAGCA |
|  |  |  | delXq25B-R4 | TCACAGACACACATGGCTCA |
| 57 | **del Xq25-F1** | **GCAGGTCAGCTTGAAACTCC** | del Xq25-F2 | GGAGCCAATAACAGCTCTGC |
|  |  |  | del Xq25-R2 | CAGAATCTGCGTGGAGAACA |
|  |  |  | del Xq25-F3 | TGCTCCAGTGATTGTCCTCA |
|  | **del Xq25-R1** | **AGGGCAGCAAGTTTCTTTCA** | del Xq25-R3 | AGGGAGATGGAGAACGTGTG |
|  |  |  | del Xq25-F4 | GCAGGTCAGCTTGAAACTCC |
|  |  |  | del Xq25-R4 | TGGAGCCTGGAATCGTAGAC |
| 58.A | **delXq25B1-F1** | **ACTGAATGCCCAATGAGAGG** |  |  |
|  | **delXq25B1-R1** | **ACAACCTTGGAGACCAATGC** |  |  |

**Table S1. Primers used for the validation of array-CGH results (in bold) and for the case-control study**

| **LOSS code** | **PRIMERS FOR FIRST STEP SCREENING** (5’→ 3’) Forward. Reverse | | **PRIMERS FOR CONFIRMATION** (5’→ 3’) Forward. Reverse | |
| --- | --- | --- | --- | --- |
| 60.A | **delXq26.3-F1** | **TGACACAAGTGTTGGGGAAA** |  |  |
|  | **delXq26.3-R1** | **TGGACAGCTGCATAGTCCTG** |  |  |
| 60.D | **delXq27.1-F1** | **TTGGAATCCTTTCTCGGATG** |  |  |
|  | **delXq27.1-R1** | **CCGGTTTTCTGAGTGACCAT** |  |  |
| 61 | **del Xq27.2-F1** | **CAGATAGGGCACTGCAGACA** | del Xq27.2-F3 | ACACCAGAGACTGGGGAGTG |
|  | **del Xq27.2-R1** | **CCTGCTTTTGCCAGGTAGAG** | del Xq27.2-R3 | CTGGTGCATTTCATGCTCTC |
| 64 | **del Xq27.3-F1** | **CGGCCAAAGTATTCTGGGTA** | del Xq27.3-F3 | ACCAACTGGTGTGGCTAAGG |
|  |  |  | del Xq27.3-R3 | CACGTGACTCTGACCAGCAT |
|  | **del Xq27.3-R1** | **CAGGGGAGGTGTGATAGCAT** | del Xq27.3-F4 | GGCACTGAAAAGATGGGAAA |
|  |  |  | del Xq27.3-R4 | GGTTTCACTGCTACCCTCCA |
| 66 | **del Xq27.3B-F3** | **TATGCACGTTGAAGCCTGAG** | del Xq27.3B-F4 | TGGCACATGGTAGGCATTTA |
|  | **del Xq27.3B-R3** | **CGAGGAAACTGAAGCCACTC** | del Xq27.3B-R4 | GGTTTAGCTCCAGGATGCAG |
| 66.A | **delXq28B-F1** | **TCCAGAGAGCCACCTTGACT** |  |  |
|  | **delXq28B-R1** | **GGGCAGAACACAAAGGACAT** |  |  |
| 67 | **del Xq28-F1** | **ACAGCATAGGCTCTGGAGGA** | del Xq28-F2 | GCCAGGCAGAAGAGTACCTG |
|  |  |  | del Xq28-R2 | CTGTGCTCCTGTTCCAGTGA |
|  | **del Xq28-R1** | **CCCTGAGCTAGGTGCTTCAC** | del Xq28-F3 | CACTGGAACAGGAGCACAGA |
|  |  |  | del Xq28-R3 | TGTGGAGCAACAGGTGAGAG |
| 69 | **del Xq28B2-F1** | **TTACAGGTGTGTGCCACCAT** | del Xq28B2-F2 | GCCAGAGGAAATGGCTTGTA |
|  |  |  | del Xq28B2-R2 | GATGGGTAGGTGGAGAACCA |
|  | **del Xq28B2-R1** | **TGTCAACTGGTTGGAACAGG** | del Xq28B2-F4 | AGGGAAATGGAAACCAAAGG |
|  |  |  | del Xq28B2-R4 | TCTGAAGGCAAAGGATCACA |
