# Supplementary material for: High Resolution X Chromosome-Specific Array-CGH Detects New CNVs in Infertile Males
Source: PLoS One. 2012 Oct 9;7(10):e44887. doi: 10.1371/journal.pone.0044887 (PMC3467283; doi:10.1371/journal.pone.0044887)
Supplement: Table S2 — List of TaqMan Copy number assay codes used for the validation process. (DOC) [file pone.0044887.s003.doc]

**Table S2. List of TaqMan Copy number assay codes used for the validation process**

|  | |
| --- | --- |
| **CNV CODE** | **TaqMan Copy Number Assay ID** |
| CNV19 | Hs00131100_cn |
| CNV20 | Hs02640673_cn |
| CNV25 | Hs05664369_cn |
| CNV26 | Hs04101901_cn |
| CNV27 | Hs04101901_cn |
| CNV30/31 | Hs04119215_cn |
| CNV39 | Hs05614245_cn |
| CNV40 | Hs04510684_cn |
| CNV55 | Hs05675355_cn |
| CNV58 | Hs05642887_cn |
| CNV60 | Hs07514404_cn |
