# Supplementary material for: High Resolution X Chromosome-Specific Array-CGH Detects New CNVs in Infertile Males
Source: PLoS One. 2012 Oct 9;7(10):e44887. doi: 10.1371/journal.pone.0044887 (PMC3467283; doi:10.1371/journal.pone.0044887)
Supplement: Table S4 — Phenotypic features according to the presence/absence of losses in patients, including the comparison between carriers and no-CNV carriers of hormonal parameters and testis volumes (A) as well as the description of patients with losses detected during both the array-CGH and case-control studies (B). (DOC) [file pone.0044887.s005.doc]

**Table S4. Phenotypic features according to the presence/absence of losses in patients, including the comparison between carriers and no-CNV carriers of hormonal parameters and testis volumes (A) as well as the description of patients with losses detected during both the array-CGH and case-control studies (B).**

| **A.** | | | |
| --- | --- | --- | --- |
|  | **Loss Carriers (n=47)** | **No-CNV Carriers (n=36)** | **p value** |
| **FSH (U/L)** |  |  |  |
| Mean Value ± SD | 12.9 ± 8.37 | 14.21 ± 10.02 | 0.712 |
| Median (25^th^-75^th^ percentile) | 11.29 (5.80 - 18.55) | 13.0 (5.20 – 21.80) |  |
| **LH (U/L)** |  |  |  |
| Mean Value ± SD | 5.28 ± 2.64 | 4.40 ± 1.85 | 0.243 |
| Median (25^th^-75^th^ percentile) | 5.22 (3.60 - 6.90) | 3.85 (3.20 – 5.62) |  |
| **TESTOSTERONE.(ng/ml)** |  |  |  |
| Mean Value ± SD | 4.78 ± 1.65 | 6.07 ± 4.50 | 0.454 |
| Median (25^th^-75^th^ percentile) | 4.57 (3.27 - 6.64) | 5.00 (3.50 - 6.90) |  |
| **TESTIS VOLUME (ml)** |  |  |  |
| Mean Value ± SD | 12.45 ± 4.57 | 9.96 ± 3.85 | 0.014 |
| Median (25^th^-75^th^ percentile) | 13.0 (9.0 - 16.0) | 10.0 (7.00-13.00) |  |

| **B.** | | | | | |
| --- | --- | --- | --- | --- | --- |
| **Patient ID** | **CNV code** | **Total sperm count (n x 10^6^)** | **FSH (U/L)** | **Mean testicular volume (ml)** | **Testis histology** |
| 05-123 | 15 | 0 | 14.1 | 15 | MA |
| 05-172 | 15 | 0 | 4.9 | 11.5 | SCOS Type2; sp+ |
| 06-111 | 15 | 0 | 18.5 | 7 | SCOS Type1; sp- |
| 06-157 | 15 | 0 | 3.7 | 18 | MA; sp+ |
| 06-201 | 15 | 0 | 13 | 7 | MA; sp- |
| 07-99 | 15 | 0 | 6.8 | 18 | SCOS Type1; sp- |
| 07-505 | 15 | 0 | 14.1 | 8 | SCOS Type1; sp- |
| 08-26 | 15 | 0 | 23.6 | 9 | SCOS Type1; sp- |
| 08-259 | 15 | 0 | 12.7 | 4.5 | SCOS Type1; sp- |
| 08-262 | 15 | 0 | 7.23 | 13 | MA + HS; sp- |
| 08-282 | 15 | 0 | 18.7 | 11.5 | SCOS Type1; sp- |
| 09-263 | 15 | 0 | 11.2 | 8 | SCOS Type1; sp- |
| 09-137 | 15 | 0 | 19 | 13 | HS |
| A833 | 15 | 0.6 | 7.68 | 14 | n.p |
| 09-233 | 15 | 0.79 | 22.8 | 11.5 | SCOS + HS |
| A456 | 15 | 2.7 | 9.8 | 10.5 | n.p |
| A688 | 15 | 6.1 | 10.16 | 8 | n.p |
| 07-13 | 16 | 0 | 10.1 | 16 | MA; sp+ |
| A828 | 17 | 20.00 | 13.3 | 11.5 | n.p |
| 07-96 | 18 | 0 | 29.9 | 13 | SCOS Type1; sp- |
| MMP718 | 22 | 6.4 | 5.3 | 14 | n.p |
| A142 | 23 | 0.01 | 2.77 | 18 | n.p |
| M4 | 24 | 0 | 26 | 0.5 | n.p |
| A630 | 31 | 7.20 | 3.94 | 23 | n.p |
| 09-126 | 31 | 8 |  | 22 | n.p |
| 08-190 | 32 | 0 | 22.7 | 5 | n.p |
| A162 | 32 | 0.01 | 8.6 | 13 | n.p |
| 07-22 | 50 | 0 | 14.6 | 15 | SCOS Type1; sp- |
| MMP550 | 54 | 0.24 | 13.1 | 8 | n.p |
| 06-188 | 56 | 0 | 15.5 | 10.5 | SCOS Type1; sp- |
| 05-238 | 57 | 0.22 | 5 | 11.5 | HS |
| 07-30 | 61 | 0 | 34 | 16 | SCOS Type1; sp- |
| 08-79 | 64 | 0 | 3.75 | 20 | MA; sp- |
| M14 | 64 | 0 | 12.5 | 9 | SCOS Type1; sp- |
| M15 | 64 | 0 | 24.3 | 5 | n.p |
| A2 | 64 | 0.01 | 2.15 | 16 | n.p |
| A616 | 64 | 0.01 | 7.2 | 19.5 | n.p |
| A1020 | 64 | 2.4 | 3.5 | 12 | n.p |
| MMP597 | 64 | 17.4 | 3.3 | 14 | n.p |
| 07-516 | 66 | 0 | 27.7 | 11.5 | SCOS + HS; sp+ |
| 05-196 | 67 | 0 | 30.7 | 16 | SCOS Type1 |
| MMP704 | 67 | 1.02 | 9.7 | 14 | n.p |
| MMP676 | 67 | 21.5 | 3.6 | 14 | n.p |
| MMP687 | 67 | 57.20 | 6.9 | 14 | n.p |
| A955 | 69 | 2.5 |  | 15 | n.p |
| A609 | 69 | 8.64 | 7.96 | 13 | n.p |
| A448 | 15,17 | 13.44 | 9.35 | 18 | n.p |
| 07-341 | 15,64 | 0 | 15 | 13 | MA; sp- |
| 08-92 | 15,69 | 0 | 23 | 10 | SCOS Type1; sp- |
| 05-205 | 15,69 | 0 | 13.2 | 13 | SCOS Type1; sp- |

| **B. continue** | | | | | |
| --- | --- | --- | --- | --- | --- |
| **Patient ID** | **CNV Code** | **Total sperm count (n x 10^6^)** | **FSH (U/L)** | **Mean testicular volume (ml)** | **Testis histology** |
| A371 | 15,69 | 0.01 | 11.38 | 16 | n.p |
| 05-236 | 15,71 | 0 | 5.8 | 10.5 | SCOS + MA |
| 07-170 | 16,69 | 0.53 | 5.8 | 15 | MA + HS |
| 07-30 | 61,69 | 0 | 34 | 14 | SCOS Type1; sp- |

Non-parametric test was performed for all comparisons, except for testis volume for which Student t-test was used. sp-/sp+: no spermatozoa/spermatozoa recovered by Testicular Sperm Extraction (TESE); n.p.: not performed. MA: Maturation Arrest; SCOS Type1: Pure SCOS; SCOS Type2: SCOS + Complete maturation in rare tubules; HS:Hypospermatogenesis
