# Supplementary material for: High Resolution X Chromosome-Specific Array-CGH Detects New CNVs in Infertile Males
Source: PLoS One. 2012 Oct 9;7(10):e44887. doi: 10.1371/journal.pone.0044887 (PMC3467283; doi:10.1371/journal.pone.0044887)
Supplement: Table S5 — Array-CGH study: comparison of semen parameters according to the number of CNVs in the control group. (DOC) [file pone.0044887.s006.doc]

**Table S5. Array-CGH study: comparison of semen parameters according to the number of CNVs in the control group**

| **CONTROLS (n=103)** | | | | |
| --- | --- | --- | --- | --- |
|  | **Sperm concentration (n x 10^6^ / ml)** | **p** | **Total sperm number (n x 10^6^)** | **p** |
| **0 CNV (n=61)** | **94.7±46.9 (88.0; 27.0-250.0)** | **0.869** | **330.7±209.5 (295.0; 39.5-957.0)** | **0.334** |
| **≥ 1 CNV (n=42)** | **95.7±53.1 (82.0; 24.0-220.0)** |  | **289.6±180.4 (269.7; 50.0-772.0)** |  |
| **0 LOSS (n=83)** | **94.4±48.2 (85.0; 27.0-250.0)** | **0.877** | **320.6±204.4 (280.0; 39.5-957.0)** | **0.653** |
| **≥ 1 LOSS (n=20)** | **97.8±54.9 (96.0; 24.0-205.0)** |  | **286.2±172.8 (254.7; 50.0-617.4)** |  |
| **0 GAIN (n=75)** | **97.1±48.4 (90.0; 25.0-250.0)** | **0.352** | **322.9±201.9 (283.5; 39.5-957.0)** | **0.339** |
| **≥ 1 GAIN (n=28)** | **89.6±52.1 (79.0; 24.0-220.0)** |  | **289.7±189.8 (269.7; 56.0-772.0)** |  |
| **≤ 1 CNV (n=92)** | **95.0±49.8 (84.5; 25.0-250.0)** | **0.785** | **318.6±202.7 (280.0; 39.5-957.0)** | **0.579** |
| **> 1 CNV (n=11)** | **96.2±47.3 (113.0; 24.0-168.0)** |  | **274.5±158.9 (269.5; 78.7-570.0)** |  |
| **≤1 LOSS(n=101)** | **95.1±49.7 (87.0; 24.0-250.0)** | **0.886** | **313.5±200.1 (271.0; 39.5-957.0)** | **0.616** |
| **>1 LOSS(n=2)** | **96.5±27.6 (96.5; 77.0-116.0)** |  | **331.9±88.3 (331.9; 269.5-394.4)** |  |
| **≤ 1 GAIN (n=99)** | **94.0±49.7 (84.0; 24.0-250.0)** | **0.129** | **317.4±201.0(280.0; 39.5-957.0)** | **0.528** |
| **> 1 GAIN (n=4)** | **123.2±31.6 (115.5; 94.0-168.0)** |  | **237.7±94.9 (229.5; 138.0-354.0)** |  |

Sperm concentration and total sperm number are expressed as: mean ± SD (median; range). Significance is depicted by a *p* value< 0.05
