# Supplementary material for: High Resolution X Chromosome-Specific Array-CGH Detects New CNVs in Infertile Males
Source: PLoS One. 2012 Oct 9;7(10):e44887. doi: 10.1371/journal.pone.0044887 (PMC3467283; doi:10.1371/journal.pone.0044887)
Supplement: Table S6 — Array-CGH study: Spermatogenic characteristics of patients and controls carrying more than one CNV. (DOC) [file pone.0044887.s007.doc]

**Table S6**. **Array-CGH study: Spermatogenic characteristics of patients and controls carrying more**

| **Patient Code** | **n of CNVs (n Losses + n Gains)** | **Sperm concentration (n x 10^6^ / ml) – Total sperm count (n x 10^6^)** | **Testis Histology** |
| --- | --- | --- | --- |
| 05-196 | 2 (1+1) | 0.00-0.00 | Pure SCOS; sp- |
| 05-205 | 2 (2+0) | 0.00-0.00 | Pure SCOS; sp- |
| 05-236 | 2 (2+0) | 0.00-0.00 | SCOS + Spermatocytic arrest; sp- |
| 06-111 | 2 (1+1) | 0.00-0.00 | Pure SCOS; sp- |
| 07-99 | 2 (1+1) | 0.00-0.00 | Pure SCOS; sp- |
| 07-341 | 2 (2+0) | 0.00-0.00 | Scleroialinosis. Spermatogonic arrest.with rare spermatocytes; sp-. |
| 07-516 | 2 (1+1) | 0.00-0.00 | 50% SCOS. Hypospermatogenesis; sp+. |
| 08-79 | 2 (1+1) | 0.00-0.00 | Spermatocytic arrest; sp- |
| 08-92 | 2 (2+0) | 0.00-0.00 | Pure SCOS; sp-. |
| 08-259 | 2 (1+1) | 0.00-0.00 | Pure SCOS; sp-. |
| M8 | 2 (0+2) | 0.00-0.00 | n.p. |
| A371 | 2 (2+0) | 0.01-0.01 | n.p. |
| A448 | 2 (2+0) | 1.80-13.44 | n.p. |
| MMP718 | 2 (1+1) | 2.00-6.4 | n.p. |
| 07-13 | 3 (1+2) | 0.00-0.00 | Maturation Arrest. |
| 05-238 | 3 (1+2) | 0.22-0.22 | Hypospermatogenesis; sp+ |
| 07-30 | 3 (2+1) | 0.00-0.00 | Pure SCOS; sp- |
| 07-170 | 3 (2+1) | 0.15-0.53 | Incomplete Spermatogenic Arrest (spermatogonia) + Hypospermatogenesis |
| A2 | 3 (1+2) | 0.01-0.01 | n.p. |
| **Control Code** | **n of CNVs (n Losses + n Gains)** | **Sperm concentration (n x 10^6^ / ml)** | **Total sperm count (n x 10^6^)** |
| 07-507 | 2 (0+2) | 94 | 188 |
| 08-119 | 2 (1+1) | 44 | 154 |
| C11 | 2 (1+1) | 31.5 | 78.7 |
| C17 | 2 (1+1) | 150 | 570 |
| C32 | 2 (1+1) | 123 | 480 |
| C35 | 2 (1+1) | 24 | 122 |
| C81 | 2 (0+2) | 118 | 354 |
| C119 | 2 (2+0) | 116 | 394.4 |
| 08-414 | 3 (0+3) | 168 | 138 |
| C53 | 3 (0+3) | 113 | 271 |
| C110 | 3 (2+1) | 77 | 269.5 |

**than one CNV.**

Testis histologies were available only for azoospermic patients. sp-/ sp+: no spermatozoa/spermatozoa recovered by Testicular Sperm Extraction (TESE); n.p.: not performed.
